# Initial experience with an 11 MeV self-shielded medical cyclotron on operation and radiation safety

**DOI:** 10.4103/0971-6203.35724

**Published:** 2007

**Authors:** G. S. Pant, S. Senthamizhchelvan

**Affiliations:** Department of Nuclear Medicine, All India Institute of Medical Sciences, New Delhi, India

**Keywords:** ^18^F-FDG, radiation monitoring, self-shielded cyclotron

## Abstract

A self-shielded medical cyclotron (11 MeV) was commissioned at our center, to produce positron emitters, namely, ^18^F, ^15^O, ^13^N and ^11^C for positron emission tomography (PET) imaging. Presently the cyclotron has been exclusively used for the production of ^18^F- for ^18^F-FDG imaging. The operational parameters which influence the yield of ^18^F- production were monitored. The radiation levels in the cyclotron and radiochemistry laboratory were also monitored to assess the radiation safety status in the facility. The target material, ^18^O water, is bombarded with proton beam from the cyclotron to produce ^18^F- ion that is used for the synthesis of ^18^F-FDG. The operational parameters which influence the yield of ^18^F- were observed during 292 production runs out of a total of more than 400 runs. The radiation dose levels were also measured in the facility at various locations during cyclotron production runs and in the radiochemistry laboratory during ^18^F-FDG syntheses. It was observed that rinsing the target after delivery increased the number of production runs in a given target, as well as resulted in a better correlation between the duration of bombardment and the end of bombardment ^18^F- activity with absolutely clean target after being rebuilt. The radiation levels in the cyclotron and radiochemistry laboratory were observed to be well within prescribed limits with safe work practice.

The inception of positron emission tomography (PET) into mainstream clinical imaging has led to a steady increase in the number of medical cyclotrons worldwide. At present, more than 350 cyclotrons are in operation worldwide as per the international atomic energy agency (IAEA) database.[1] There has been steady increase in the number of cyclotrons installed both in developed as well as developing nations over the past decades. Most of these cyclotrons are being utilized to produce ^18^F-FDG, either for own use at the center or for distribution to other PET centers.[1] Many commercial vendors have also installed cyclotrons to produce and distribute ^18^F-fluorodeoxyglucose (^18^F-FDG) to PET centers in their vicinity.[2,3] The ultra short-lived radionuclides used in PET warrant the presence of a cyclotron in the vicinity for their production. In a cyclotron-PET facility, the radiation safety needs to be more stringent due to penetrating gamma radiation and high specific gamma ray constant for positron emitters.[4–7] Despite the availability of adequate shielding in the cyclotron and radiochemistry laboratories, occupational workers remain skeptical about radiation exposure from positron emitters.

The competent authority Atomic Energy Regulatory Board (AERB) made regulatory inspections before approving the facility for routine clinical use. More than 4,000 patients have been benefited with PET/CT imaging. Since the self-shielded medical cyclotron installed at our center was the first of its kind in India, various radiation safety issues were to be addressed at the time of commissioning both from regulatory as well as operational standpoint. This paper describes operational and radiation safety aspects in a self-shielded medical cyclotron.

## Materials and Methods

### Medical cyclotron

The radioisotope delivery system (RDS) Eclipse^RD^ (CTI, Knoxville, TN, now Siemens Medical Solutions) is a self-shielded medical cyclotron, installed in the basement of our PET facility. It is a fixed-energy isochronous cyclotron, capable of accelerating protons (H^-^) to 11 MeV energy, with dual-beam extraction option.[8] The cyclotron vault area is 6.7 m × 6.9 m with a ceiling height of 3.6 m. The ceiling and walls of the cyclotron vault are made of 0.5 m thick concrete with a density of 2,480 kg m^−3^. The operator's station is outside the cyclotron vault.

### Cyclotron operation

The cyclotron has targetries to produce ^18^F, ^11^C, ^13^N, ^15^O and ^18^F_2_ gas under automated control. So far, our cyclotron has been exclusively used to by produce ^18^F^-^ for the synthesis of ^18^F-FDG. The ^18^F^-^ ions are produced by the ^18^O(p, n)^18^F nuclear reaction in a silver target assembly filled with 1.25 ml of enriched ^18^O water.

The radionuclide production is started after about 30 min of initialization. During this period, radio frequency system may be tuned with varying operating potential (RF conditioning). The typical operational parameters of the cyclotron are given in Table 1. The production yield of ^18^F^-^ depends on various factors such as enrichment of ^18^O water, target volume, target current, energy of the beam, variation in argon pressure on the target, bombardment duration, number of previous bombardments on the target (target status), status of the delivery lines from target to the radiochemistry laboratory. Most of these parameters were optimized during the initial runs of the cyclotron and operated at fixed values as given in Table 1 for all production runs. Apart from the parameters mentioned in Table 1, one important parameter, which influences the end of bombardment (EOB) yield, is the rinsing of the target with high-purity water immediately after the delivery of the activity from cyclotron target to radiochemistry laboratory. The effect of target rinsing was observed in 292 production runs. Out of the 292 production runs, 142 were done without rinsing the target and 150 were done with target rinsing immediately after delivering the activity to the chemistry synthesis box. Though the cyclotron has two beam lines, we have not yet tried to produce ^18^F^-^ simultaneously on both the beam lines. Instead, both the beam lines were used alternatively for all ^18^F^-^ production. The radiochemistry laboratory is equipped with an Explora FDG_4_ chemistry module, mini cell and hot cell with robotic arms for remote handling of radioisotopes and dispensing individual patient doses.

**Table 1:** Cyclotron operational parameters[8]

*Parameters*	*Typical value*
Main magnet current	∼230.38 A
Main magnetic offset	∼2.53–3.52 A
Dee voltage	34–34.5 kV
High vacuum	∼3.8–4.7×10^−6^ Torr
RF frequency	∼72.42 MHz
Ion source current	0.11–0.15 A
Bias voltage	14 kV
Bias current	∼3.88 mA
Foil transmission efficiency	70-78%
Beam strength	40 μA

### Radiation monitoring

#### Cyclotron operation

**Figure 1:**
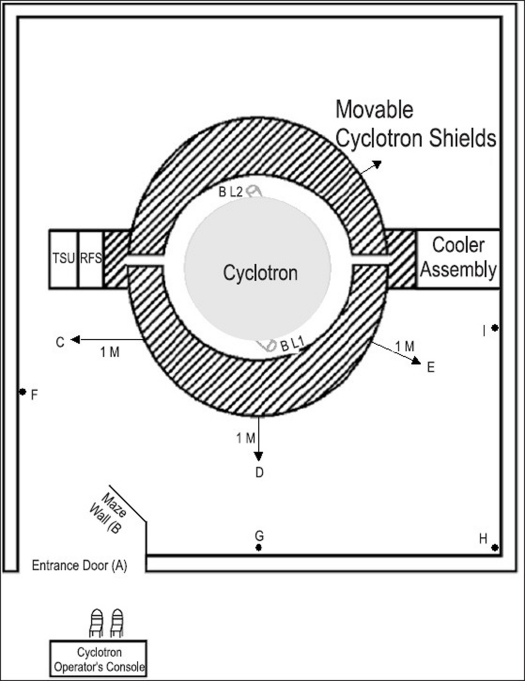
Layout of cyclotron room with points of measurements for radiation survey

The gamma dose levels in the cyclotron vault and radiochemistry laboratory were monitored with a calibrated RAM GAM-1 portable gamma ray survey meter (Rotem Industries, Israel). Victoreen 190N portable neutron survey meter (Fluke Biomedical, Ohio, U.S.A.) was used to monitor the secondary neutrons around the cyclotron shield. The radiation monitoring in the vicinity of cyclotron was done at specific locations (point A to I). Figure 1 shows the layout of cyclotron room with points of measurements for radiation survey. Point A was located at the entrance of the cyclotron room, points B through E were located inside the cyclotron room at 1 m from the surface of cyclotron shield and points F through I were located on the inner wall of the cyclotron room on the first beam line (BL1) side. When the second beam line (BL2) was used, point B through E and point F through I were measured on the other side of the cyclotron room at respective locations. The radiation survey for gamma rays and the neutrons were done at the above-mentioned points before, during and immediately after the cyclotron production runs. During cyclotron operation, the entrance door is locked to avoid any unauthorized entry; furthermore, there is a beam ‘on’ indicator for caution. Environmental radiation survey was also done at the outlet of the exhaust systems of both cyclotron and radiochemistry laboratory.

### Target rebuild

When the yield of ^18^F^-^ production decreases after several production runs or otherwise, the ^18^F silver target assembly needs to be cleaned and rebuilt. The main source of radiation exposure during target rebuild was the havar foil (target window), which forms the entrance of proton beam into a highly pressurized ^18^O-enriched water target used for the production of ^18^F^-^. Havar is an alloy containing cobalt, chromium, iron, nickel, manganese, tungsten and molybdenum.[9] The havar foil gets exposed both to an intense primary beam of protons and to a large flux of secondary neutrons, produced by the ^18^O(p, n)^18^F reaction. As the havar foil needs periodic replacement, it constitutes a solid radioactive waste whose handling, storage and disposal must be done very carefully. They need to be stored as solid radioactive waste in shielded container for at least 2 years.[9] The radiation monitoring was carried out during the target cleaning and rebuild process.

### Radiochemistry laboratory

The radiation monitoring was done in the radiochemistry laboratory just before the delivery of bolus to the FDG synthesis box, during synthesis and after the delivery of ^18^F-FDG from synthesis box to the production vial in the hot cell. The radiation levels were also measured outside the hot cell during individual dose dispensing. The emissions from radiochemistry synthesis box (mini cell) and hot cell are measured at the outlet of radiochemistry exhaust system.

## Results

### Cyclotron operation

**Figure 2:**
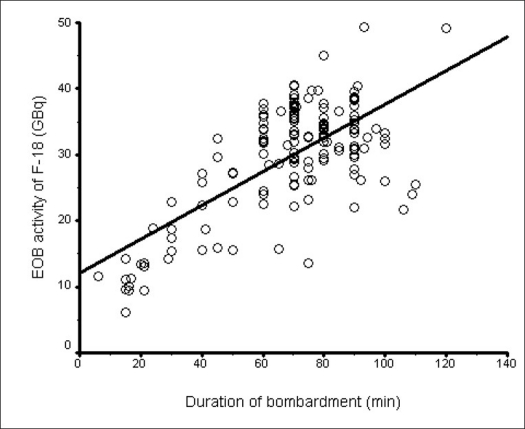
Correlation between the duration of bombardment and ^18^F^-^ production yield in all production runs with target rinsing

**Figure 3:**
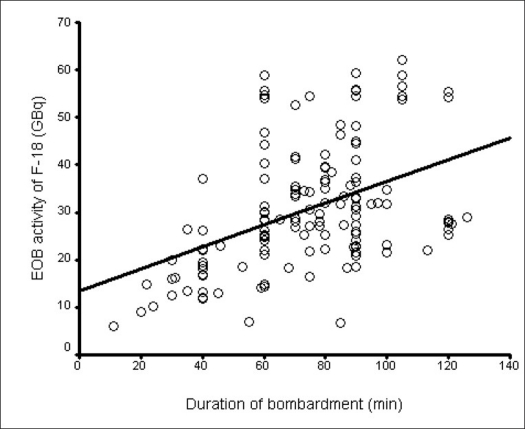
Correlation between the duration of bombardment and ^18^F^-^ production yield in all production runs without target rinsing

So far, we have made more than 400 ^18^F production runs. In some of the runs, the activity was directly delivered to the radiochemistry synthesis module. In 292 runs, the activity was measured in a dose calibrator in the hot cell before being transferred to the radiochemistry synthesis module. The mean duration of bombardment in 292 production runs on the ^18^F^-^ target was 71.42 ± 23.92 min, with the average yield of ^18^F^-^ being 30.10 ± 10.83 GBq (813.5 ± 292.7 mCi), as shown in Table 2. The effect of target rinsing was observed in 292 production runs. Out of the 292 production runs, 142 were done without rinsing the target – the mean duration of bombardment on the ^18^F^-^ target was 74.60 ± 24.50 min, with the average yield of ^18^F- being 30.66 ± 12.86 GBq (828.6 ± 347.6 mCi); and 150 were done with rinsing the target – the mean duration of bombardment on the ^18^F^-^ target was 68.37 ± 23.03 min, with the average yield of ^18^F^-^ being 29.57 ± 8.51 GBq (799.2 ± 230 mCi). The correlations between the duration of bombardment and ^18^F^-^ production yield in production runs with and without rinsing the target are shown in Figures 2 and 3 respectively. Pearson's product moment correlation test showed better correlation (r = 0.69, *P* < 0.001) between the duration of bombardment and ^18^F^-^ production yield when the target was rinsed as compared to no target rinse (r = 0.44, *P* < 0.001). Independent Student's ‘t’ test also showed that rinsing the target significantly improves yield of ^18^F^-^ as compared to no rinse (*P* < 0.001). The average ^18^F^-^ yield was found to be 26 GBq/h (703 mCi/h) with rinsing and 24.7 GBq/h (668 mCi/h) without rinsing the target at the end of production run. Table 3 shows the effect of target rinse on the longevity of target usage per target rebuild. When the target was rinsed, the target usage per rebuild was 1709.20 μAh as compared to no target rinse with 470.85 μAh. Thus only rinsing of the target itself increased the μAh on an average by a factor of 3.6. The yield of ^18^F^-^ depended mostly on the status of the targetry in a given beam line. With a newly rebuilt target and recently replaced delivery line between the cyclotron and radiochemistry laboratory, the yield has always been satisfactory. Generally on a typical run, the target was irradiated for about 60-90 min depending upon the yield obtained on the previous run and the requirement for the day.

**Table 2:** Details of ^18^F^-^ production runs

*Parameter*	*Total production runs (n=292)*		*Without target rinsing (n=142)*		*With target rinsing (n=150)*	
			
	*Duration of bombardment (min)*	*Total activity GBq (mCi)*	*Duration of bombardment (min)*	*Total activity GBq (mCi)*	*Duration of bombardment (min)*	*Total activity GBq (mCi)*
Mean ± SD	71.40 ± 23.92	30.10 ± 10.83	74.60 ± 24.51	30.66 ± 12.86	68.37 ± 23.03	29.57 ± 8.51
		(813.5 ± 292.7)		(828.6 ± 347.6)		(799.2 ± 230)
Minimum	6.00	6.07	11.00	6.11	6.00	6.07
		(164)		(165)		(164)
Maximum	126.00	62.04	126.00	62.04	120.00	49.26
		(1677)		(1677)		(1331)
Total	20849.00	8790.06	10594.00	4353.76	10255.00	4436.30
		(237569)		(117669)		(119900)

**Table 3:** Effect of target rinsing on target longevity

*Classification of ^18^F^-^ production runs*	*No. of runs*	*Total duration of bombardment (h)*	*No of target rebuild*	*No. of μAh per rebuild*
All production runs	292	347.48	19	731.54
Without target rinsing	142	176.52	15	470.85
With target rinsing	150	170.92	4	1709.20

### Radiation levels in the cyclotron vault

The gamma and neutron dose rates measured at the locations of interest in the vicinity of cyclotron are given in Table 4. These dose rates are average of measurements done during 79 production runs of ^18^F^-^. During the production runs, the gamma dose rate near the entrance door (point A) was 1.5 ± 0.85 μSv/h and the neutron dose rate was 0.01 ± 0.001 μSv/h. The gamma and neutron dose rates at 1 meter from the outer surface of the cyclotron shield (points B through E) in the cyclotron vault varied from 3.1 ± 1.3 μSv/h to 50.7 ± 2.8 μSv/h and from 0.03 ± 0.01 μSv/h to 14.2 ± 1.1 μSv/h respectively. The neutron dose rate was not in the detectable range just before and after the production run in the cyclotron vault.

The radiation levels in the cyclotron vault near the inside walls (points F through I) during the production run varied from 3.2 ± 1.2 μSv/h to 15.8 ± 5.3 μSv/h for gamma, and the neutron dose rates varied from 3.5 ± 0.6 to 10.2 ± 2.5 μSv/h. The measured dose rates at the inside walls of cyclotron room were almost as per the manufacturer's specification (≤20 μSv/h). During the cyclotron operation, the exposure rates were measured at the outlet of both the cyclotron and radiochemistry exhausts located on the terrace, which varied from 0.2 mSv/h to 1.5 mSv/h.

### Radiation dose rates during target rebuild

On opening the cyclotron shields 24 h after bombardment, the dose levels near the target carousel varied from 70 mSv/h to 100 μSv/h. The havar foil that forms the target window is highly radioactive and gives exposure rates ≥8 mSv/h at contact while removing from the target for cleaning and rebuild. High radiation dose rates were observed only during the target cleaning and rebuild due to havar foil. However, removal of havar foil takes only few seconds and needs to be done by experienced workers.

### Radiation dose rates in the radiochemistry laboratory

The gamma dose rates before receiving the ^18^F^-^ activity from the cyclotron and after the completion of ^18^F-FDG synthesis ranged from 0.02 to 0.08 μSv/h, which was nearly the background level. During ^18^F-FDG synthesis in the Explora module, the dose rates around the synthesis box varied from 1.2 to 8.4 μSv/h at various locations. The exposure levels around the hot cell ranged from 0.15 to 0.72 μSv/h during the ^18^F-FDG dose dispensing, against the limit of 10 μSv/h in controlled area. An exposure rate of 10 μSv/h with 40 working hours a week and 50 weeks a year results in an annual exposure limit of 20 mSv (averaged over 5 years) to whole body as prescribed by the International Commission on Radiological Protection (ICRP) and the AERB.[10]

### Occupational exposure to staff

The staff working both in cyclotron and the radiochemistry laboratory is covered under the national personnel monitoring service (PMS) using CaSO_4_: Dy thermoluminescence dosimetry (TLD) badges. Radiation dose to occupational workers in cyclotron and radiochemistry laboratory as measured by the PMS available for 12 months has been shown in Table 5. The dose received by occupational workers in cyclotron is less than 5% of annual dose limit (20 mSv) to the whole body and less than 2% of annual limit (500 mSv) to the extremities. Similarly, the doses received by occupational workers in the radiochemistry laboratory are less than 10% of annual limit to the whole body and less than 1% of the annual limit to the extremities. This is well expected as all the operations in cyclotron and radiochemistry laboratory are completely automated with adequate shielding.

**Table 4:** Measurement of gamma and neutron dose rates in the cyclotron vault

*Measurement point*	*Gamma dose rate before cyclotron run (μSv/h)*	*Gamma dose rate during cyclotron run (μSv/h)*	*Gamma dose rate immediately after cyclotron run (μSv/h)*	*Neutron dose rate during cyclotron run (μSv/h)*
A	0.6 ± 0.4	1.5 ± 0.8	0.5 ± 0.07	0.01 ± 0.001
B	0.8 ± 0.3	3.1 ± 1.3	1.2 ± 0.4	0.03 ± 0.01
C	0.8 ± 0.5	50.7 ± 2.8	5.2 ± 0.8	14.2 ± 1.1
D	0.7 ± 0.2	15.7 ± 4.5	3.7 ± 1.3	5.8 ± 0.8
E	0.5 ± 0.3	25.6 ± 3.2	5.4 ± 1.6	6.5 ± 1.6
F	0.4 ± 0.1	5.4 ± 0.1	0.4 ± 0.2	3.5 ± 0.6
G	0.3 ± 0.2	15.8 ± 5.3	1.3 ± 0.4	10.2 ± 2.5
H	0.3 ± 0.2	3.2 ± 1.2	0.5 ± 0.2	6.4 ± 0.5
I	0.3 ± 0.2	12.7 ± 2.3	0.7 ± 0.3	9.3 ± 3.8

**Table 5:** Radiation dose to occupational workers in cyclotron and radiochemistry laboratory as measured by national personnel monitoring service using CaSO_4_: Dy TLD badges (Jan-Dec 2006)

*Occupational workers (OW) in Cyclotron/ radiochemistry laboratory*	*Whole body dose in mSv*	*Extremity (Wrist) dose in mSv*
OW1 (Cyclotron)	0.35	7.95
OW2 (Cyclotron)	0.85	0.45
OW3 (Radiochemistry)	0.60	4.45
OW4 (Radiochemistry)	1.8	3.4

## Discussion

The medical cyclotrons dedicated to produce positron-emitting radionuclides are increasing in medical institutions/hospitals due to the well-established role of PET imaging in clinical practice. However, the knowledge and experience of operating these installations is limited in our country as they have been in use only for the past few years. Our experience of working with an 11 MeV cyclotron is nearly 2 years now. We have been able to optimize the operating conditions for the cyclotron to get the maximum yield with a good number of production runs after the target rebuild. Though the production yield of ^18^F^-^ depends on various factors such as enrichment of ^18^O water, target volume, target current, energy of the beam, etc., most of these parameters are fixed for all production runs. In our experience, we have observed that rinsing of the target immediately after production runs significantly improves the production yield and longevity of target usage per target rebuild. This also reduces the repeated target-rebuild process, which involves radiation exposure to personnel performing the target rebuild. However, one learns to optimize these factors with experience of working with the system.

Timely replacement (monthly) of delivery lines that carry the activity from cyclotron target to the hot cell in the radiochemistry laboratory is an essential component of maintenance that helps in getting good yield. Rinsing the target with water (HPLC grade) immediately after each production run has been extremely helpful in keeping the target clean and increasing the number of production runs for a given target.

The radiation safety issues in a cyclotron-PET facility are much different from the conventional nuclear medicine facilities because of penetrating gamma photons of 511 keV, higher specific gamma ray constant of positron emitters and secondary neutrons from the cyclotron during production. Therefore, the work practice for occupational workers in a cyclotron-PET facility has to be more stringent.[5,7] In the present study, we have monitored the dose rates in the cyclotron room, radiochemistry laboratory to check the radiation safety status in the facility. The dose rates in these areas were found to be well within acceptable limits. The neutron and gamma dose rates measured in this study were comparable with similar studies on the same systems available in the literature.[11,12] The dose rates due to secondary neutrons always remained well within the recommended limits around the shields during beam operation for the maximum beam current in use and for the longest duration of production run, indicating that the shields adequately minimize the exposure from neutrons in the cyclotron room. The intensity of the gamma rays and secondary neutron field produced by a cyclotron primarily depend on the beam strength. We had constantly used 40 mA beam current in all production runs.

The radiation shield of the cyclotron is composed of lead, boron, concrete and epoxy, which provides acceptable dose levels during beam operation. To ensure further radiation safety to the staff, the entrance door of the cyclotron vault was locked during beam operation so that entry into the high radiation field is strictly restricted.

Once the activity is transferred from the cyclotron to the synthesis box in the radiochemistry laboratory, the dose levels were slightly increased in the radiochemistry laboratory around the mini cell or hot cell where the activity had been delivered, and the same in cyclotron room were reduced. The dose levels around the mini and hot cell were minimal due to heavy shielding. The hot cell and mini cell could be opened only the next day when the radiation levels were negligibly small.

It is essential to monitor dose rates in the cyclotron room for personnel safety and in the exhaust system for environmental safety. If the cyclotron needs to be opened for the maintenance of ion source, target window or vacuum windows, etc., it is advisable to wait for at least 24 h after the production run to allow for the physical decay of short-lived radionuclides. The direct bombardment of internal structures and target assembly by the accelerated charged particles and neutrons leads to the generation of activation products. Amongst the possible activation reactions, the most prominent are ^63^Cu(n, α)^60^Co, ^54^Fe(n, p)^54^Mn and ^27^Al(n, p)^24^Na. The ^60^Co emits two gamma photons of average energy 1.25 MeV with a half life of 5.27 years, ^54^Mn emits 835 keV photons with a half life of 312 days and ^24^Na emits 2.75 MeV photons with a half life of 15 h.[8] Hence extreme caution needs to be taken during maintenance and repairing of the cyclotron internal components.

The occupational exposure to the staff working in the cyclotron and radiochemistry laboratory has been less than 10% of the prescribed annual dose limit to the whole body and less than 5% of the annual dose limit to the extremities, as can be seen in Table 5. The only chance of occupational exposure to the staff working in PET or PET/CT imaging is where PET radiopharmaceuticals are injected into the patients by the physicians and scanning is done by the technologists. In our recent study, it was observed that with safe work practice, the dose to occupational workers in PET/CT was only 3% of the prescribed annual dose limits.[13] One has to monitor the dose levels at various locations and measure the occupational exposures in the facility to ensure safe work practice.

## Conclusion

We have optimized the ^18^F^-^ production yield with our initial experience of working with an 11 MeV self-shielded medical cyclotron. The radiation monitoring of the cyclotron facility during the cyclotron production runs at various locations in and around the cyclotron vault, and the radiochemistry laboratory has shown that the gamma and neutron levels were well within acceptable limits. The attenuation provided by the cyclotron shields, the room walls and the distance between the source of radioactivity and the operator's location ensure that the radiation safety of occupational workers is adequately taken care of. With the developments in cyclotron design and proper planning of a cyclotron-PET facility, the radiation exposure to the staff can be further reduced.
